# Revealing propionate metabolism-related genes in glioblastoma and investigating their underlying mechanisms

**DOI:** 10.3389/fonc.2025.1529369

**Published:** 2025-04-17

**Authors:** Yuchen Sun, Huijuan Wang

**Affiliations:** ^1^ Department of Neurosurgery, The Second Hospital of Hebei Medical University, Shijiazhuang, Hebei, China; ^2^ Department of Neurology, The Second Hospital of Hebei Medical University, Shijiazhuang, Hebei, China

**Keywords:** glioblastoma, propionate metabolism, single cell analysis, prognosis, biomarker

## Abstract

**Background:**

Propionate metabolism may affect tumor growth and aggressiveness, but the role of propionate metabolism-related genes (PMRGs) in glioblastoma (GBM) remains poorly understood.

**Methods:**

Differentially expressed PMRGs (DE-PMRGs) were identified by comparing differentially expressed genes (DEGs) between GBM and normal tissues using TCGA-GBM, GSE42669, GSE162631 datasets. Functional enrichment analysis of DE-PMRGs was performed, followed by univariate Cox regression and least absolute shrinkage with selection operator (LASSO) analysis to identify potential prognostic biomarkers. In addition, prognostic models were developed and validated using independent cohorts. Genomic enrichment analysis (GSEA) was used to assess immune-related pathways in different risk subgroups. Finally, biomarker expression was confirmed using quantitative reverse transcription polymerase chain reaction (qRT-PCR).

**Results:**

Differential expression analysis identified a total of 180 DE-PMRGs, which were strongly associated with drug response and insulin signaling pathways. Six biomarkers (SARDH, ACHE, ADSL, PNPLA3, MAPK1 and SREBF2) were identified to be associated with prognosis. The accuracy of the prognostic model was confirmed using the GSE42669 dataset, with risk score and MGMT promoter status identified as independent prognostic factors. GSEA showed enrichment of immune response activation and cell cycle regulatory pathways. qRT-PCR validation showed up-regulation of PNPLA3 and SARDH, and down-regulation of ADSL, in tumor tissues.

**Conclusions:**

This study identified six PMRGs (SARDH, ACHE, ADSL, PNPLA3, MAPK1 and SREBF2) as potential prognostic biomarkers for glioblastoma. These biomarkers reveal the role of propionate metabolism in the progression of glioblastoma and may serve as important indicators of patient prognosis and treatment strategies.

## 1 Introduction

Glioblastoma (GBM) is a highly aggressive brain tumor classified as WHO grade IV, characterized by its severe malignancy, high recurrence rates, and unfavorable prognosis (1). Currently, the standard treatment for GBM involves surgical removal of the tumor, followed by adjuvant radiotherapy and chemotherapy (2). Even with these treatments, the median survival time for GBM patients is under 15 months, and the five-year survival rate is still below 5% (3, 4). It remains a great challenge to assess the prognosis of GBM and adopt more effective therapeutic measures to improve patients’ survival time and quality of life. Therefore, exploring the regulatory mechanisms of GBM progression and potential prognostic markers is crucial for evaluating prognosis and improving treatment outcomes for GBM.

Cellular metabolism is a process of breakdown and buildup of nutrients in the cell, but also embodies the characteristics and functions of the cell (5). Moreover, metabolic reprogramming has emerged as a key hallmark of tumors. Metabolic enzymes and their byproducts play significant roles in the initiation and progression of tumors (6, 7). Amino acid metabolic reprogramming plays a key role in gliomas (8). Studies show that reprogramming amino acid metabolism not only promotes glioma cell proliferation but also enhances tumor stress tolerance by influencing signal transduction and altering epigenetic states. Additionally, metabolic changes in specific amino acids contribute to immune evasion and chemotherapy resistance in gliomas. Given that both amino acid metabolism and propionate metabolism are critical components of the complex tumor metabolic network, further exploration of propionate metabolism-related genes in glioblastoma (GBM) holds significant scientific importance. Propionate metabolism is crucial for normal physiological processes in the body, and its disruption can contribute to the development of various diseases. Research indicates that disturbances in propionate metabolism result in the accumulation of methylmalonic acid (MMA) in cells and tumors, which enhances the invasiveness of tumor cells (9). A study by Ana P. Gomes and colleagues utilized the triple-negative breast cancer (TNBC) 4T1 cell line to develop a model for lung metastasis in breast cancer. Results suggested that changes in propionate metabolism may affect the progression and aggressiveness of cancer, and further affect the anticipated outcomes for patients (10). Additionally, genes associated with propionate metabolism (PMRGs) may be linked to the prognosis of patients with hepatocellular carcinoma (HCC) (11). However, the role of PMRGs in the pathogenesis of GBM remains unclear.

This study investigates the genes involved in propionate metabolism in GBM and develops a risk prediction model based on six specific genes to assess their functional significance and prognostic potential. Additionally, we conducted a thorough analysis of the relationship between this prognostic model, immune cell infiltration, and somatic mutations. Additionally, scRNA-seq data were integrated to identify cell clusters associated with key prognostic genes in GBM. We also validated the expression of these six genes in tumor and adjacent tissues using quantitative reverse transcription polymerase chain reaction (qRT-PCR) *in vitro*. These findings could contribute to personalized diagnostics, enhance prognostic predictions, and inform treatment decisions for patients with GBM.

## 2 Materials and methods

### 2.1 Data source

The TCGA-GBM dataset (https://www.cancerimagingarchive.net/collection/tcga-gbm/) comprises RNA sequencing data from 144 GBM samples, of which 141 include survival information, along with 5 normal samples. This dataset served as the training cohort. The GSE42669 dataset was obtained from the Gene Expression Omnibus (GEO) database (https://www.ncbi.nlm.nih.gov/geo/). This dataset (GPL6244) contains RNA sequencing data from 58 GBM tissue samples, each with corresponding survival data, and was used as the validation cohort. We then identified 604 PMRGs from the GeneCards database with a relevance score exceeding 7 (see **Supplementary Table S1**). Information for the single-cell dataset GSE162631 was acquired from the TISCH database.

### 2.2 Identification of DEGs

The DEGs between GBM and normal groups were identified using the DESeq2 package (version 3.44.3) (12) in the TCGA-GBM dataset, with a significance threshold of P value < 0.05, baseMean > 50, and |log2FC| > 1. The results of the differential analysis were visualized using a volcano plot created with the ggplot2 package (version 3.3.5) (13). The expression levels of the top 100 DEGs between the two groups were presented using a heatmap.

### 2.3 Screening and functional enrichment of PMRGs

DE-PMRGs were identified by overlapping DEGs with PMRGs. Following this, Gene Ontology (GO) and Kyoto Encyclopedia of Genes and Genomes (KEGG) enrichment analyses for the DE-PMRGs were performed using the clusterProfiler package (version 3.16.0) (14), applying a significance threshold of P < 0.05.

### 2.4 Screening for biomarkers and construction of risk model

The 141GBM samples were divided into a training cohort of 99 samples and an internal testing cohort of 42 samples, following a 7:3 ratio. A univariate Cox regression analysis (15) was conducted on the DE-PMRGs to identify possible candidate genes. Subsequently, LASSO analysis was conducted using the identified candidate genes. The selected genes were then used as biomarkers for this study. Patients were categorized into high-risk and low-risk groups based on the optimal thresholds derived from the risk scores calculated using the biomarkers. Riskscore = $\sum_{\text{l}}^{\text{n}}\text{coef}\left(\text{genei}\right)*\text{expression}\left(\text{genei}\right)$ . And the K-M survival curves were drawn. The survivalROC package (version 1.0.3) (16) was used to draw the Receiver Operating Characteristic (ROC) curves, assessing the model’s predictive accuracy. Correlation analyses between clinicopathological factors and the risk model in the TCGA training cohort were conducted using chi-square tests. The risk model was validated using additional cohorts, including the internal testing cohort and the GSE42669 dataset.

### 2.5 Independent prognostic analysis

Independent prognostic analyses were conducted on clinical characteristics, including IDH mutation status and MGMT promoter methylation, as well as the risk score in the TCGA training cohort.

### 2.6 Clinical features and survival analysis

First, the risk scores were compared across various clinical subgroups. Next, survival analysis was conducted using the survival package (version 3.4-0) (17) to evaluate different clinical characteristics within the two risk subgroups, with Kaplan-Meier curves generated to illustrate the findings.

### 2.7 Functional enrichment of two risk subgroups

The GSEA enrichment analysis was performed on two risk subgroups by the clusterProfiler software package (v 3.16.0) (14) (|NES| > 1, NOM P < 0.05). Finally, the top 10 results for GO and KEGG significance were visualized individually.

### 2.8 Mutation analysis

The somatic mutation dataset was sourced from the GDC database. Mutational differences among various risk subgroups were analyzed using the maftools package (v 2.4.10) (18). The 15 genes with the highest mutation frequencies were visualized through waterfall plots. In addition, the pathways with high frequency of mutational oncogenic pathways in two risk subgroups were screened and visualized using the OncogenicPathways package.

### 2.9 Immune microenvironment analyses

The stromal score, immune score, ESTIMATE score, and tumor purity were calculated for the two subtypes using the ESTIMATE package (version 1.0.13). Next, the abundance of 64 cell types in each sample of the TCGA-GBM dataset was evaluated using Xcell (http://xCell.ucsf.edu/). The results were presented using box plots and heatmaps for better visualization. Finally, the relationship between these cell types and risk scores was examined. The MCP-counter algorithm (http://github.com/ebecht/MCPcounter) was utilized to impute the content of the 10 cell types (8 immune cells, fibroblast and epithelial cell) between the two risk subgroups. The results were shown by heatmap, box plot and radar plot. Finally, the relationship between the 10 cells and risk scores was illustrated in the correlation lollipop plot.

### 2.10 Single-cell RNA seq analysis

This study utilized the GSE162631 single-cell dataset for data annotation. Clustering results were visualized using UMAP techniques (19). Following this, biomarker expression was analyzed within individual cell clusters, with the findings illustrated through clustering plots and heatmaps.

### 2.11 Expression validation

In the TCGA-GBM dataset, the expression differences of biomarkers between GBM and normal groups were compared using the rank-sum test. The results were presented by box-plots.

### 2.12 Quantitative real-time polymerase chain reaction

In this study, a total of 5 pairs samples of patients from the Second Hospital of Hebei Medical University were utilized qRT-PCR, including 5 GBM tumor samples and 5 control tissue samples. The Ethics Committee of the Second Hospital of Hebei Medical University approved the study (2024-AE160), and all patients provided informed consent.

First, the total RNA of the frozen control and tumor sample was extracted a by TRIzol reagent, after which RNA detection was performed based on the NanoPhotometer N50. Then, an equal amount of mRNA was reverse transcribed to synthesize cDNA by SureScript-First-strand-cDNA-synthesis-kit, and the cDNA was diluted 5-20 times with ddH2O (RNase/DNase free). Next, the qPCR reaction was performed with 2 x Universal Blue SYBR Green qPCR Master Mix kit according to the following reaction system: predenaturation at 95°C for 1 min; Denatured 20s at 95°C, annealed 20s at 55°C, extended 30s at 72°C; 40 cycles. Finally, the gene expression level was calculated by the 2^-ΔΔCT^ method. Primer sequences for the genes can be seen in **Supplementary Table S2**.

### 2.13 Statistical analysis

All bioinformatics analyses were undertaken in R language. And the Spearman was employed to perform the correlation analysis.

## 3 Results

### 3.1 Screening and functional enrichment of DE-PMRGs

**Figure 1:**
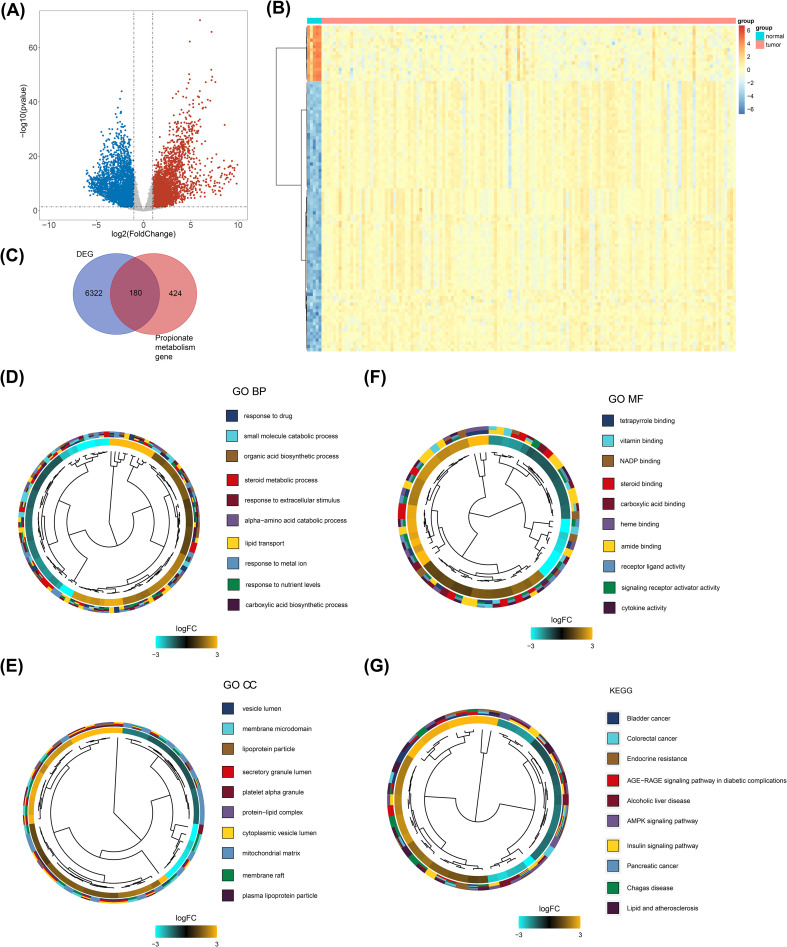
Screening and functional enrichment of DE-PMRGs. **(A)** Gene volcano plot of tumor vs normal samples. Red dots indicate that the gene expression is up-regulated, blue dots indicate that the gene expression is down-regulated, and gray dots indicate that there is no significant difference in these genes. **(B)** Heatmap of tumor vs normal sample top100 differential genes. Each small square represents each gene, and its color indicates the expression level of that gene, the higher the expression level the darker the color (red is high expression, blue is low expression). **(C)** Venn diagram of the intersection of differential genes and propionate metabolism genes. **(D-F)** Clustering diagram of GO-BF, CC, and MF enrichment systems. Different colors indicate different entries enriched to. **(G)** Clustering diagram of KEGG enrichment system. Different colors indicate different entries enriched to.

A total of 6502 DEGs between the GBM and normal groups were gained, including 3446 up-regulated genes and 3056 down-regulated genes (**Figure 1A**, **Supplementary Table S3**). Of these, the expression of the top 100 DEGs was displayed in **Figure 1B**. Then, 180 DE-PMRGs (ABHD5, AGTR1, ALOX5, AR, BRCA1, CCL5 and so on) were screened by overlapping 6502 DEGs and 604 PMRGs (**Figure 1C**, **Supplementary Table S4**). Among the 180 differentially expressed propionate metabolism - related genes (DE - PMRGs), a total of 108 genes were up - regulated and 72 genes were down - regulated. The results of the enrichment analysis revealed that the DE-PMRGs implicated 1612 GO-BP entries, 55 GO-CC entries, 142 GO-MF entries and 143 KEGG pathways. The enrichment results of DE-PMRGs showed that GO-BP entries were mainly involved in the response to drug, steroid metabolic process, etc.; GO-CC entries were mainly involved in the vesicle lumen, mitochondrial matrix, etc.; GO-MF entries were mainly involved in the amide binding, signaling receptor activator activity, etc. (**Figures 1D–F**, **Supplementary Table S5**). KEGG enrichment result included insulin signaling pathway, AMPK signaling pathway, etc. (**Figure 1G**, **Supplementary Table S6** **).**

### 3.2 Biomarkers screening and risk model

**Figure 2:**
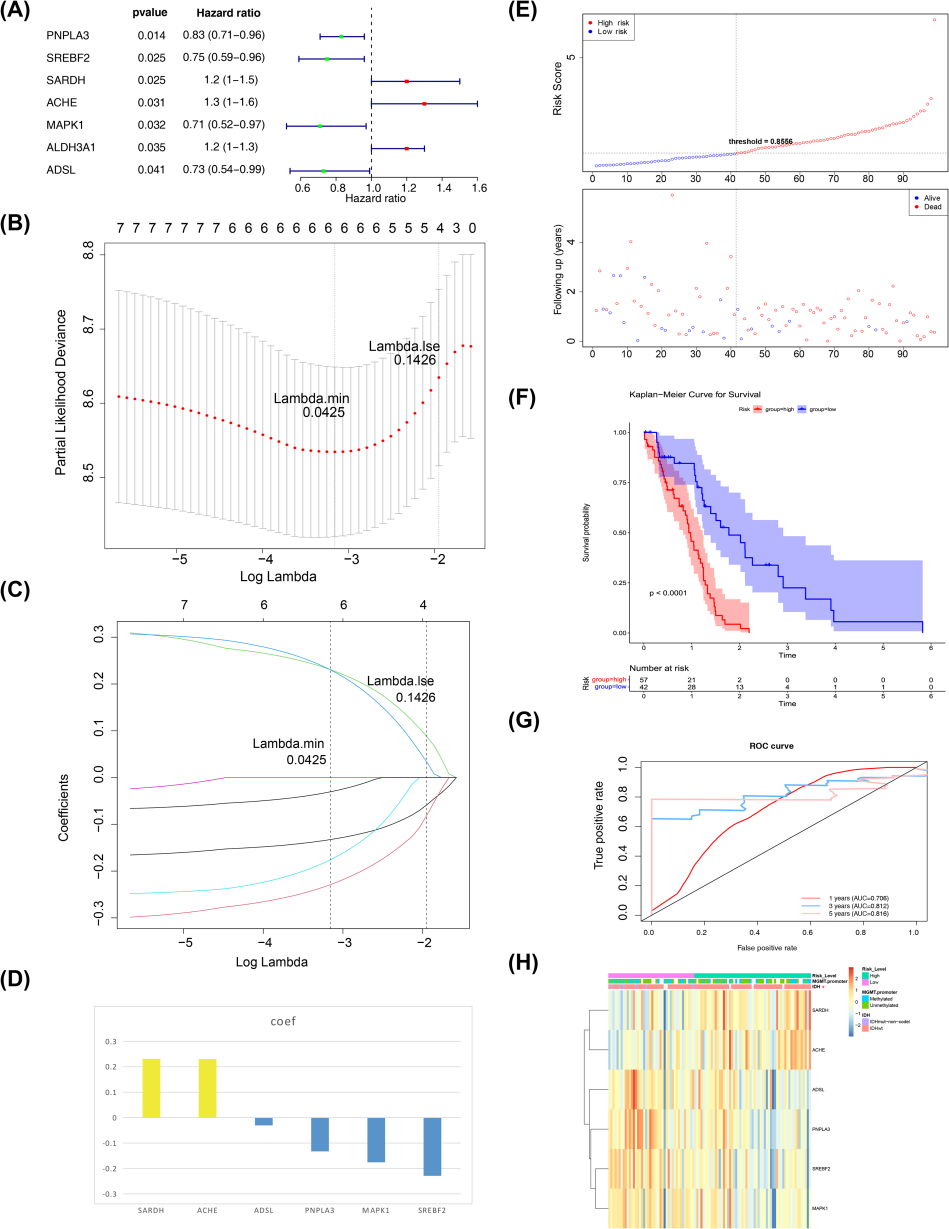
Biomarkers screening. **(A)** Forest plot of one-way cox results. The left side represents genes and their corresponding P and HR values; the red squares on the right side indicate HR values greater than 1, the green squares indicate HR values less than 1, and the lines on either side of the squares are the 95% confidence limits for the HR values. **(B)** LASSO logistic regression coefficient penalty plot. The horizontal coordinate deviance indicates the proportion of residuals explained by the model, showing the relationship between the number of characterized genes as a function of the proportion of explained residuals (dev), and the vertical coordinate is the coefficients of the genes. **(C)** LASSO Logic Coefficient Penalty Plot. The horizontal coordinate is log(Lambda) and the vertical coordinate represents the cross-validation error. As the penalty coefficients lambda are varied, the coefficients of most of the variables are finally compressed to 0, and the most lambda value is selected at the minimum of the 10-fold cross-validation error. **(D)** Penalty coefficient histogram, yellow bars indicate HR greater than 1, blue bars indicate HR less than 1. **(E)** Risk curves and scatter plots for high and low risk groups. Blue color indicates low risk group and red color indicates high risk group. **(F)** KM survival curve for Risk score. The vertical coordinate of the graph indicates the survival rate and the horizontal coordinate indicates the total survival time. The red curve indicates the high-risk group and the blue curve indicates the low-risk group. **(G)** 1, 3, and 5 year ROC curves to assess risk modeling. Pink indicates 1 year, blue indicates 3 years, and red indicates 5 years. **(H)** Correlation profiles of riskscore with each clinical trait. The top of the heatmap represents the different clinical traits, and in the heatmap, each small square represents each gene, and its color indicates the size of the expression of that gene, the larger the expression the darker the color.

A total of six biomarkers related to propionate metabolism were identified: SARDH, ACHE, ADSL, PNPLA3, MAPK1, and SREBF2 (**Figures 2A–C**). **Figure 2D** displays the penalty coefficients for these six biomarkers. Based on an optimal threshold of 0.8556, patients were categorized into high-risk (n = 57) and low-risk groups (n = 42) (**Figure 2E**). Survival analysis indicated that the low-risk group from the training set exhibited a higher survival rate (**Figure 2F**). ROC curve analyses demonstrated that the model exhibited good predictive performance, with AUC values exceeding 0.70 for 1-, 3-, and 5-year survival rates (**Figure 2G**). Correlation analysis revealed a significant link between the risk score and the coding status of IDH (**Table 1**, **Figure 2H**).

**Table 1:** Correlation analysis of risk score and IDH coding status.

Risk-Level				
	Total	High	Low	P-value
	(N=73)	(N=41)	(N=32)	
IDH				
IDHmut-non-codel	6 (8.2%)	0 (0%)	6 (18.8%)	0.0137
IDHwt	67 (91.8%)	41 (100%)	26 (81.3%)	
MGMT.promoter				
Methylated	30 (41.1%)	15 (36.6%)	15 (46.9%)	0.518
Unmethylated	43 (58.9%)	26 (63.4%)	17 (53.1%)	

**Figure 3:**
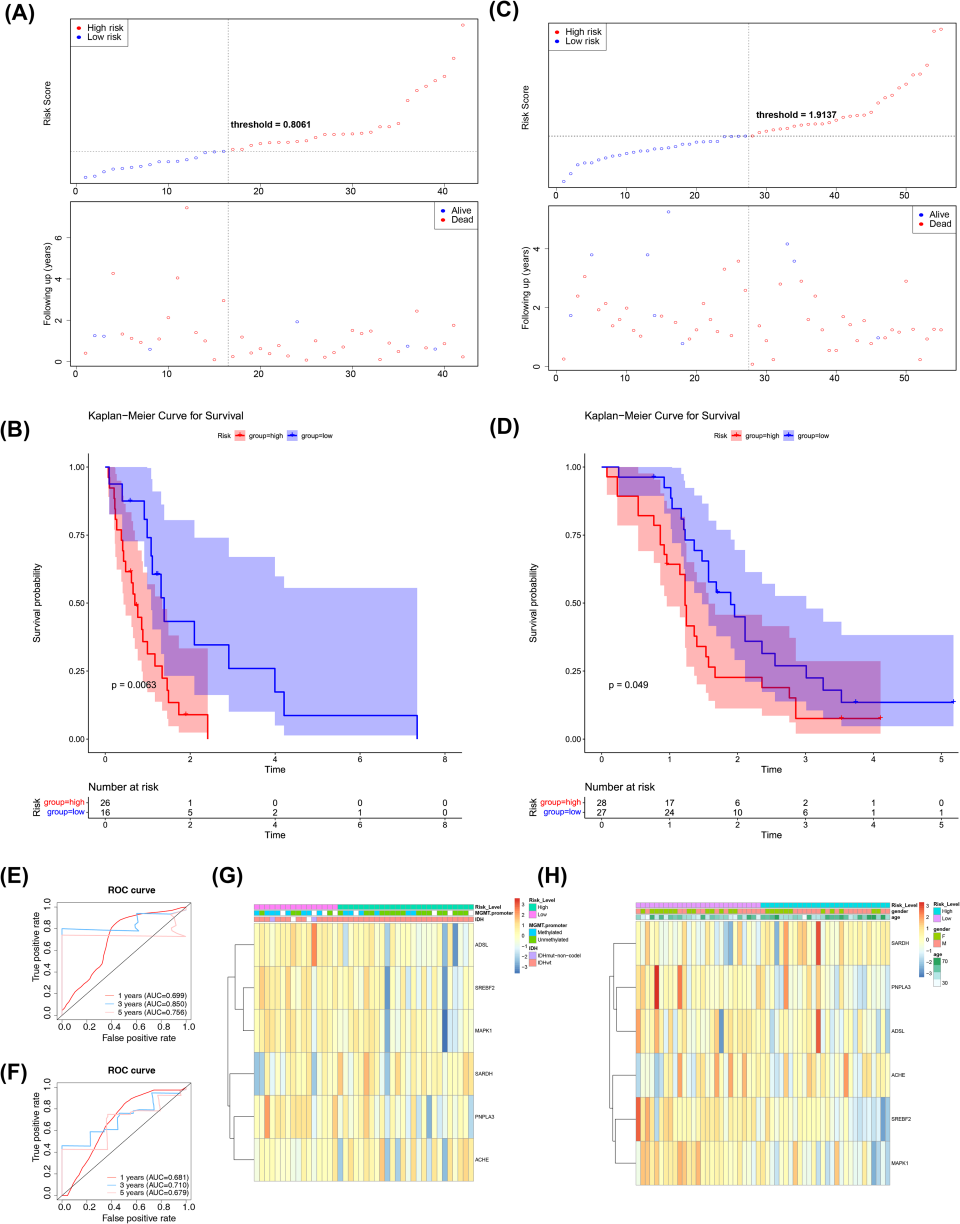
Risk model construction and validation. **(A)** Test Sets - Risk Curves for High and Low Risk Groups. Blue color indicates low risk group and red color indicates high risk group. **(B)** KM survival curve for test set-Risk score. The vertical coordinate of the graph indicates the survival rate and the horizontal coordinate indicates the total survival time. The red curve indicates the high-risk group and the blue curve indicates the low-risk group. **(C)** Validation Set - Risk Curves for High and Low Risk Groups. Blue color indicates low risk group and red color indicates high risk group. **(D)** KM survival curve for validation set-Risk score. The vertical coordinate of the graph indicates the survival rate and the horizontal coordinate indicates the total survival time. The red curve indicates the high-risk group and the blue curve indicates the low-risk group. **(E)** Test Set-ROC Curves for Assessing Risk Model Validity. Red indicates one year, blue indicates three years, and pink indicates five years. **(F)** Validation Set-ROC Curves for Assessing Risk Model Validity. Red indicates one year, blue indicates three years, and pink indicates five years. **(G)** An overview of the correlation between the test set-riskscore and each clinical trait. The different clinical traits are indicated above the heatmap. In the heatmap, each small square represents each gene, and its color indicates the magnitude of the gene’s expression, with the larger the expression the darker the color. **(H)** An overview of the correlation between the Validation set-riskscore and each clinical trait. The different clinical traits are indicated above the heatmap. In the heatmap, each small square represents each gene, and its color indicates the magnitude of the gene’s expression, with the larger the expression the darker the color.

**Figure 4:**
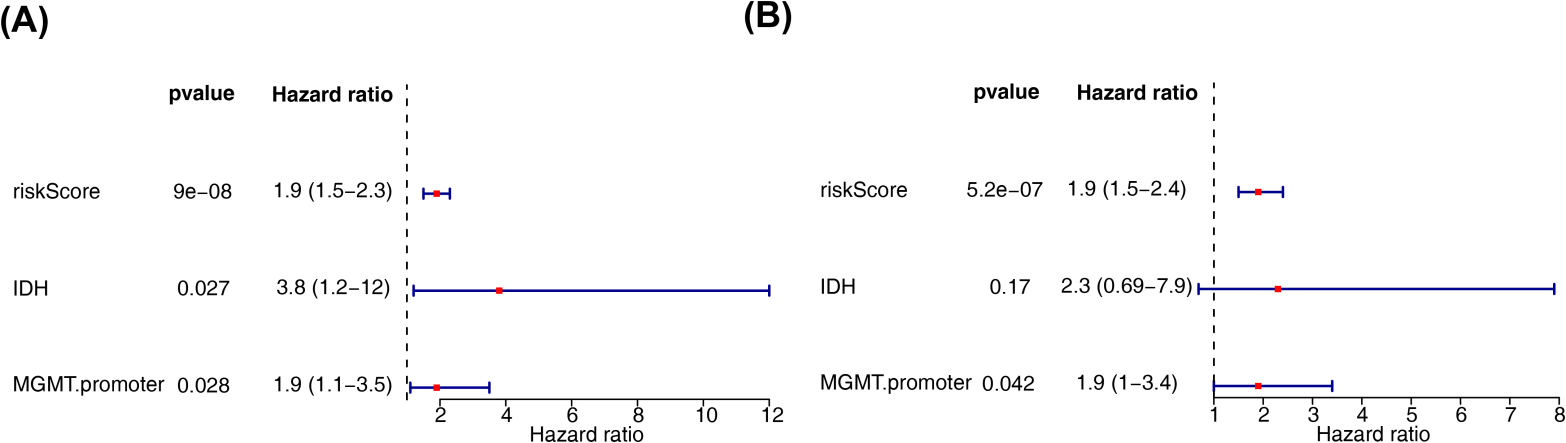
Independent prognostic analysis of risk models. **(A)** Forest plot of independent prognostic-univariate cox results. The left side represents genes and the corresponding P and HR values; the red squares on the right side indicate HR values greater than 1, the green squares indicate HR values less than 1, and the lines on either side of the squares are the 95% confidence intervals for that HR value. **(B)** Forest plot of independent prognostic-multifactorial cox results. The left side represents genes and their corresponding P and HR values; the red squares on the right side indicate HR values greater than 1, and the green squares indicate HR values less than 1, and the lines on both sides of the squares are the 95% confidence intervals of the HR values. The lines on both sides of the squares are the 95% confidence intervals of the HR values.

Next, we employed an internal testing cohort and an external validation cohort to assess the model’s predictive performance. The risk profile plots and survival curves from the validation cohorts confirmed consistency with the training set results (**Figures 3A–D**). Additionally, the AUC values for the validation cohorts were all above 0.65 (**Figure 3E–F**). In the internal testing cohort, risk scores showed a significant correlation with tumor grade (P < 0.05), whereas in the validation cohort, there was no correlation with clinical traits (**Tables 2**, **3**, **Figures 3G, H**). Ultimately, two significant factors were identified through analytical screening: the risk score and the MGMT promoter (**Figures 4A, B**).

**Table 2:** Correlation of risk scores with tumor grade in internal testing.

Risk-Level				
	Total	High	Low	P-value
	(N=32)	(N=22)	(N=10)	
IDH				
IDHmut-non-codel	1 (3.1%)	0 (0%)	1 (10.0%)	0.681
IDHwt	31 (96.9%)	22 (100%)	9 (90.0%)	
MGMT.promoter				
Methylated	13 (40.6%)	6 (27.3%)	7 (70.0%)	0.0584
Unmethylated	19 (59.4%)	16 (72.7%)	3 (30.0%)	

**Table 3:** Correlation of risk scores with tumor grade in validation cohorts.

Risk-Level				
	Total	High	Low	P-value
	(N=55)	(N=28)	(N=27)	
Age (years)				
<=60	40 (72.7%)	17 (60.7%)	23 (85.2%)	0.0829
>60	15 (27.3%)	11 (39.3%)	4 (14.8%)	
Gender				
F	25 (45.5%)	11 (39.3%)	14 (51.9%)	0.506
M	30 (54.5%)	17 (60.7%)	13 (48.1%)	

### 3.3 Clinical characterization and survival analysis between two risk subtypes

**Figure 5:**
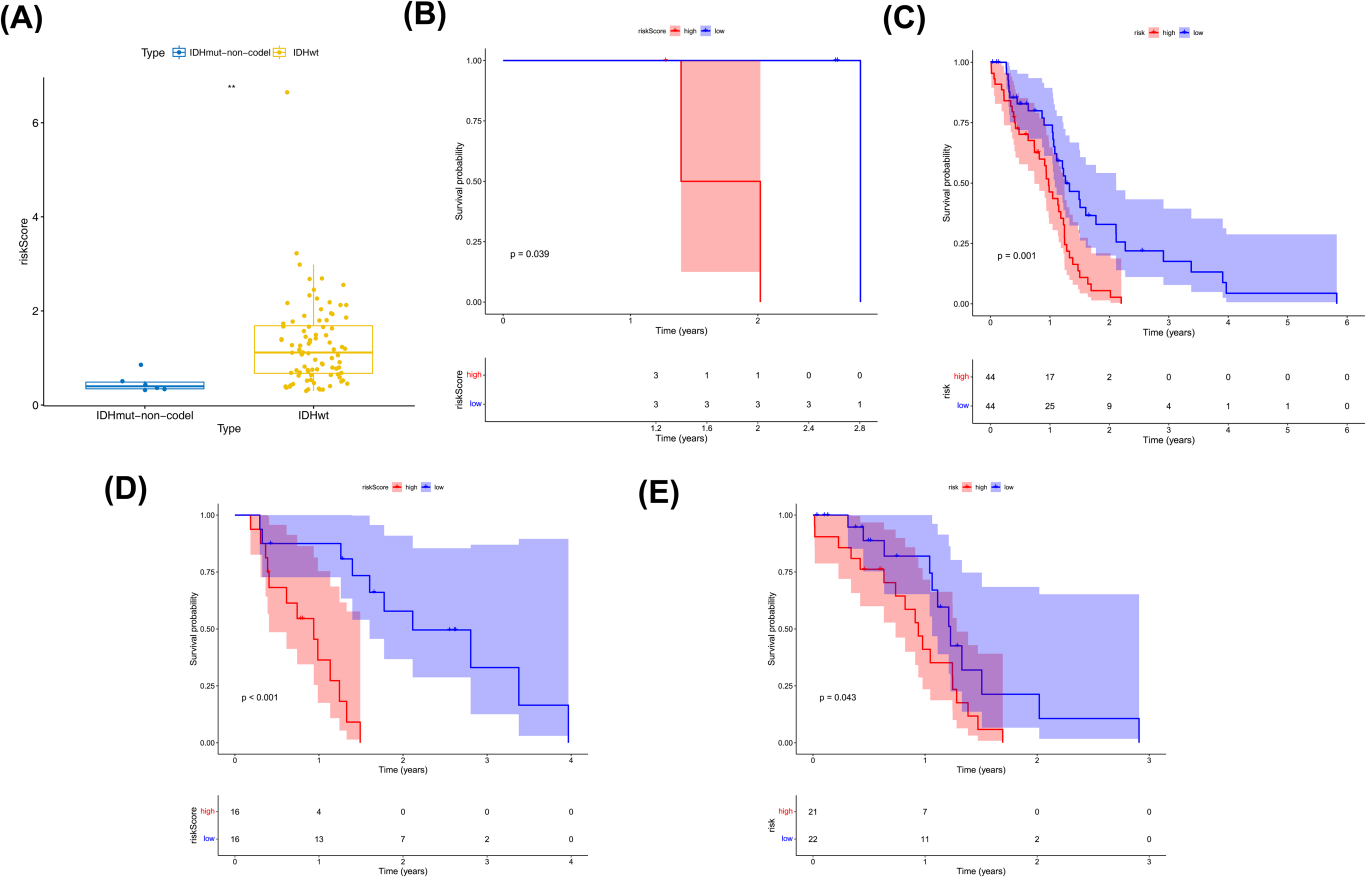
Clinical characterization and survival analysis between two risk subtypes. **(A)** Correlation of risk models with IDH traits. where the horizontal coordinates represent the different IDH subgroups and the vertical coordinates represent the values at risk. **(B, C)** IDH-KM (**B**: IDHmut,**C**: IDHwt) survival curves. Blue color indicates low risk group and red color indicates high risk group. **(D, E)** MGMT.promoter-KM (**D**:Methylated, **E**:Unmethylated) survival curves. Blue color indicates low risk group and red color indicates high risk group.

Clinical characteristic analysis revealed a significant difference in risk scores between IDH subgroups (**Figure 5A**), while no significant difference was observed among MGMT promoter subgroups (**Supplementary Figure S1**). Survival analysis demonstrated significant differences in survival rates between the two risk subgroups across all clinical categories (**Figures 5B–E**).

### 3.4 Analysis of biological processes in two risk subgroups

**Figure 6:**
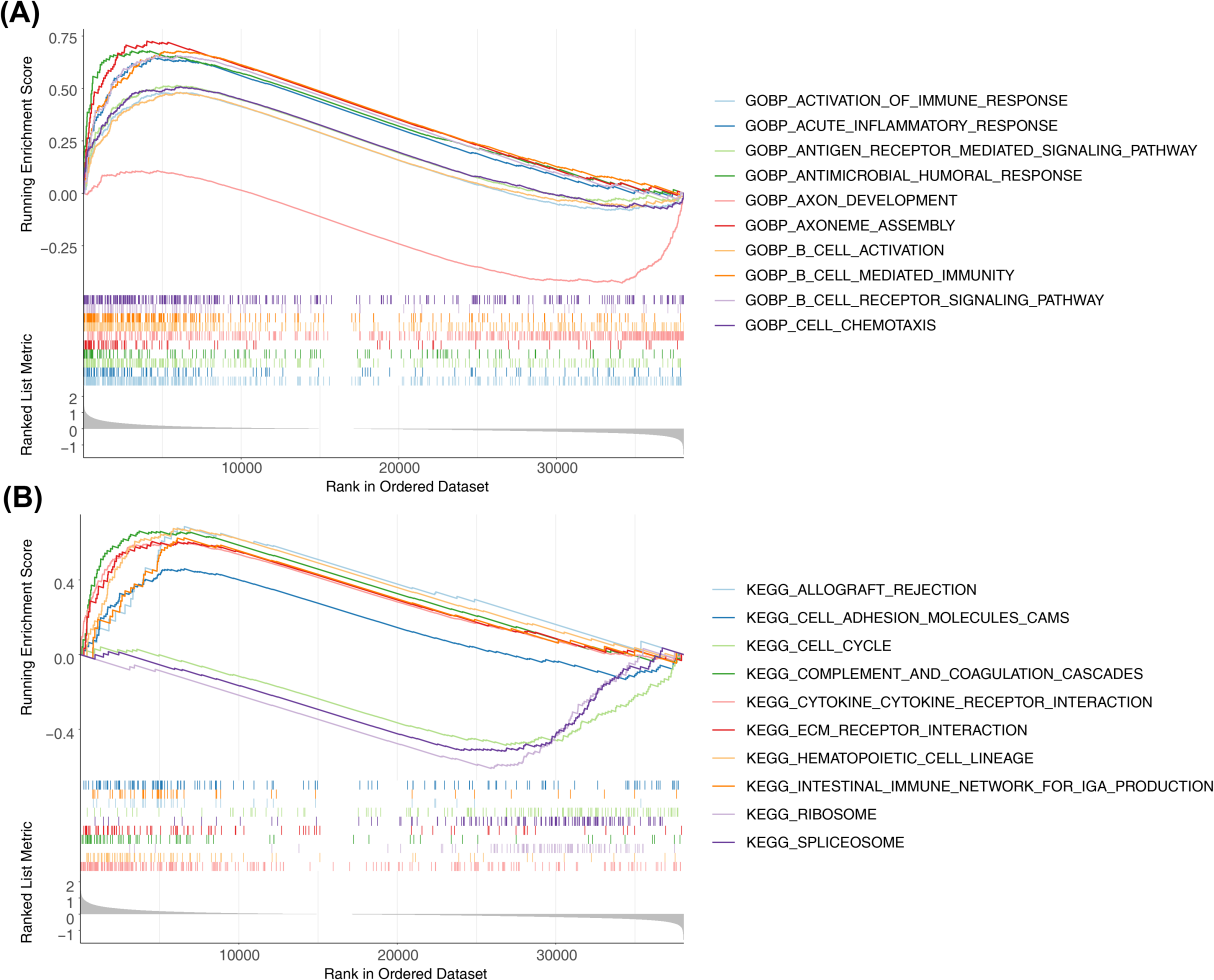
Differential analysis and enrichment analysis of high-risk and low-risk groups. **(A)** Top10-GO pathways enriched by high and low grouping. Show top10 GO paths. **(B)** Top10-KEGG pathways enriched by high and low grouping. Show top10 KEGG paths.

GSEA was performed to explore the regulatory pathways and molecular functions of the two risk subgroups. GO terms were mainly enriched to such as activation of immune response (NES=2.02), acute inflammatory response (NES=2.4), etc. (**Figure 6A**, **Supplementary Table S7**). KEGG mainly enrichment results included cell cycle (NES= -1.78), ECM receptor interaction (NES=2.12), etc. (**Figure 6B**, **Supplementary Table S8**).

### 3.5 Mutation analysis of two risk subgroups

**Figure 7:**
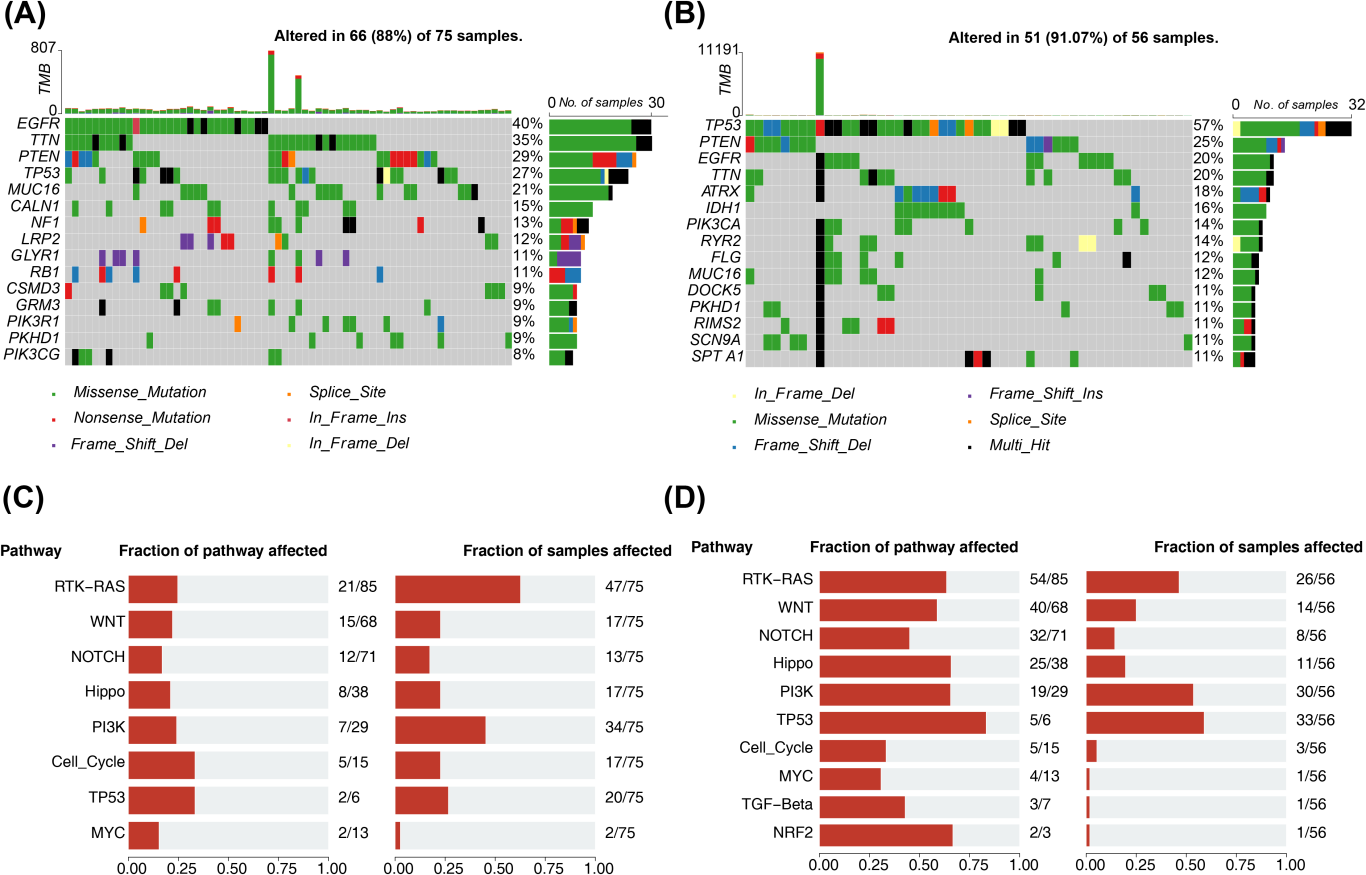
Mutation analysis of two risk subgroups. **(A)** Waterfall map of the top15 most mutated genes in the high-risk group. Different colors indicate different mutation profiles. **(B)** Waterfall plot of the top15 most mutated genes in the low risk group. Different colors indicate different mutation profiles. **(C)** Frequency of mutational oncogenic pathways in high-risk groups. Fraction of pathway affected on the left and Fraction of samples affected on the right. **(D)** Frequency of mutational oncogenic pathways in low risk groups. Fraction of pathway affected on the left and Fraction of samples affected on the right.

The mutation outcomes for the different risk subgroups were illustrated via waterfall plots respectively. Then, **Figure 7A** illustrated the 15 mutated genes in the former high-risk group, including EGFR (40%), TTN (35%), PTEN (29%), TP53 (27%), MUC16 (21%), CALN1 (15%), NF1 (13%), LRP2 (12%), GLYR1 (11%), RB1 (11%), CSMD3 (9%), GRM3 (9%), PIK3R1 (9%), PKHD1 (9%), and PIK3CG (8%). Next, 15 mutated genes were analyzed in the low-risk group, including TP53 (57%), PTEN (25%), EGFR (20%), TTN (20%), ATRX (18%), IDH1 (16%), PIK3CA (14%), RYR2 (14%), FLG (12%), MUC16 (12%), DOCK5 (11%), PKHD1 (11%), RIMS2 (11%), SCN9A (11%), and SPTA1 (11%) (**Figure 7B**). In the two risk groups, the top 8 mutational oncogenic pathways in terms of frequency included the RTK-RAS, WNT, NOTCH, Hippo, PI3K signaling pathway, and so on (**Figures 7C, D**).

### 3.6 Immune-related analysis between two risk subgroups

**Figure 8:**
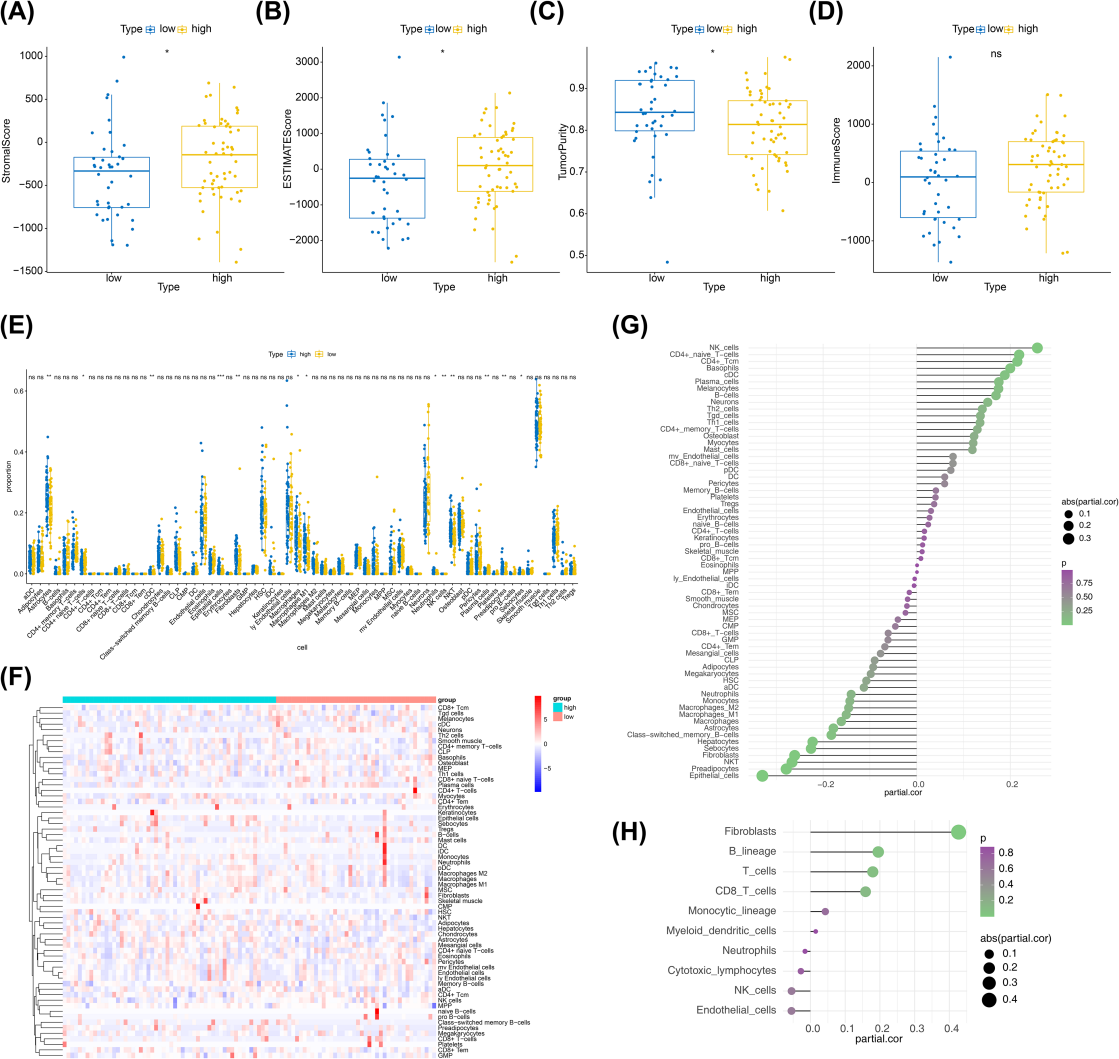
Immune-related analysis between two risk subgroups. **(A)** Box plot of immunization scores, with yellow indicating high-risk groups and blue indicating low-risk groups. **(B)** Box plots of the matrix scores, with yellow indicating the high-risk group and blue indicating the low-risk group. **(C)** Box plots of the ESTIMATE scores, with yellow indicating the high-risk group and blue indicating the low-risk group. **(D)** Box plot of tumor purity, with yellow indicating the high-risk group and blue indicating the low-risk group. **(E)** Boxplot of xcell derived cell content between high and low groups. Yellow color indicates low risk group and blue color indicates high risk group. **(F)** Xcell extrapolation of heat maps for different cell contents. Each small square represents the content of a different cell in each sample, and its color indicates how much content it contains; the more content the redder the color, and the less content the bluer the color. **(G)** Xcell derives the correlation between cell content and risk values. The horizontal coordinates of the graph indicate correlation values ranging from -1 to 1, the vertical coordinates indicate cells, the color of the bubbles indicates the significance level, and the size of the bubbles indicates the absolute size of the correlation. **(H)** MCP-counter derives the correlation between cell content and risk value. The horizontal coordinates of the graph indicate correlation values ranging from -1 to 1, the vertical coordinates indicate cells, the color of the bubbles indicates the significance level, and the size of the bubbles indicates the size of the absolute value of the correlation.

The differential analysis demonstrated that there were significant differences in stromal scores, ESTIMATE scores and tumour purity between risk subgroups (**Figures 8A–C**), and no differences for immune score (**Figure 8D**). Of these, stromal and ESTIMATE scores were lower in the low-risk group and tumour purity was lower in the high-risk group. The Xcell analysis revealed a total of 13 differential cells (astrocytes, naive CD4^+^ T cells, cDC, epithelial cells and so on) between the two risk subgroups (**Figures 8E, F**). In addition, the results of the correlation analysis illustrated that there were 10 cells (basophils, naive CD4^+^ T cells, CD4^+^ Tcm, epithelial cells, fibroblasts, hepatocytes, NK cells, NKT, preadipocytes, and sebocytes) significantly associated with the risk score (**Figure 8G**). The correlation analysis based on the MCP-counter algorithm illustrated that only fibroblasts were significantly associated with risk scores (**Figure 8H**). The remaining results were presented in **Supplementary Figure S2**.

### 3.7 Expression analysis of biomarkers in different cell types

**Figure 9:**
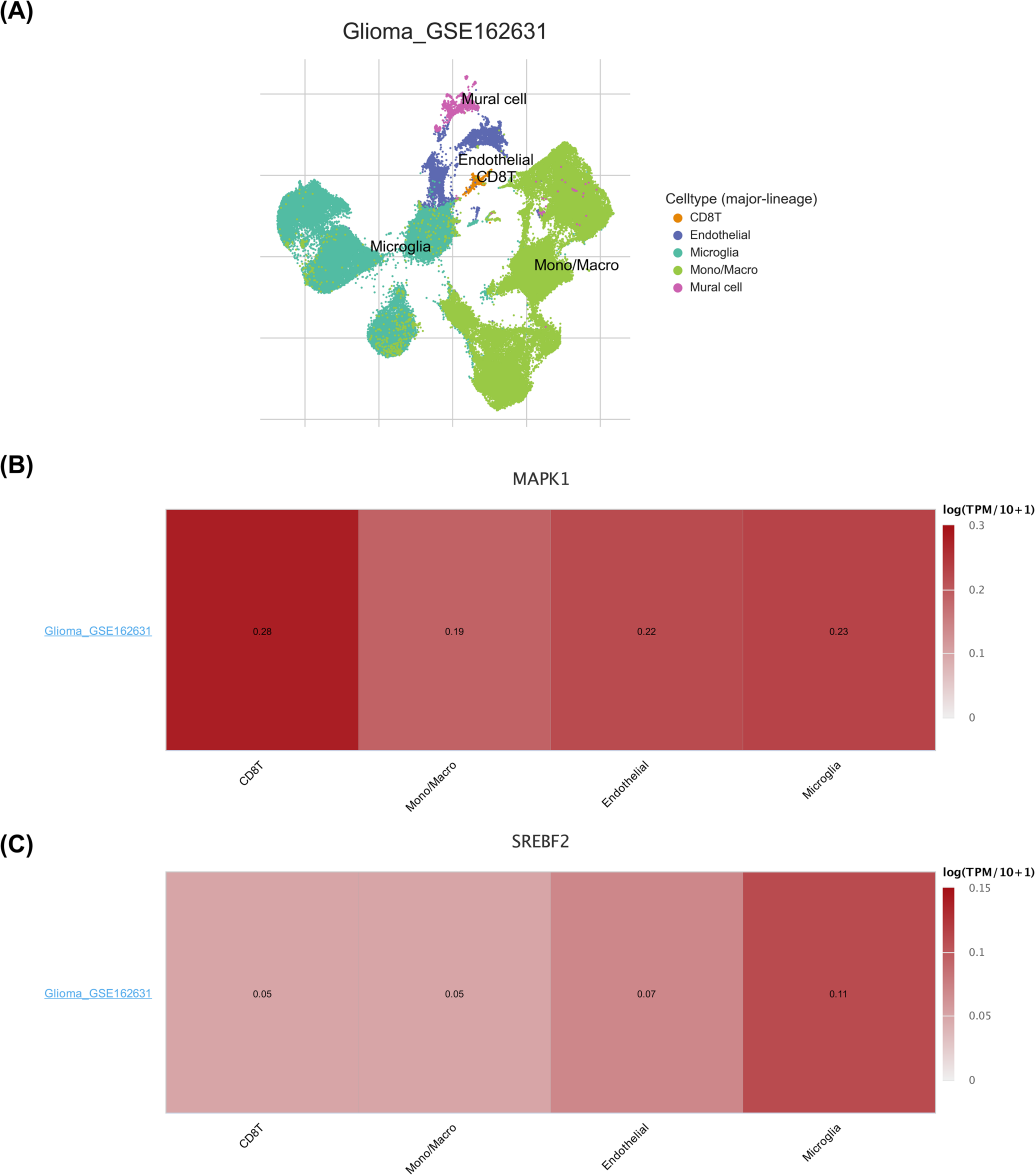
Expression analysis of biomarkers in different cell types. **(A)** Annotation status of the GSE162631 single-cell dataset. Different colors indicate different cells annotated to. **(B)** Heatmap of MAPK1 expression in the GSE162631 single-cell dataset. Darker color Y indicates higher expression. **(C)** Heatmap of SREBF2 expression in the GSE162631 single-cell dataset. Darker color Y indicates higher expression.

The cells were classified into 5 cell types (CD8 T cell, endothelial cell, microglia cell, Mono/Macro cell, and mural cell), and the distribution were visualized using UMAP (**Figure 9A**). MAPK1 was expressed in CD8 T cell, endothelial cell, microglia cell and Mono/Macro cell, with the highest expression in CD8 T cell (**Figure 9B**). The expressed of SREBF2 was highly in microglia cell (**Figure 9C**). Expression analysis showed that the expression levels of SARDH, ACHE, ADSL and PNPLA3 in each type of cells were almost zero (**Supplementary Figure S3A**). This was consistent with the results of the clustering charts (**Supplementary Figure S3B**).

### 3.8 Expression validation of biomarkers

**Figure 10:**
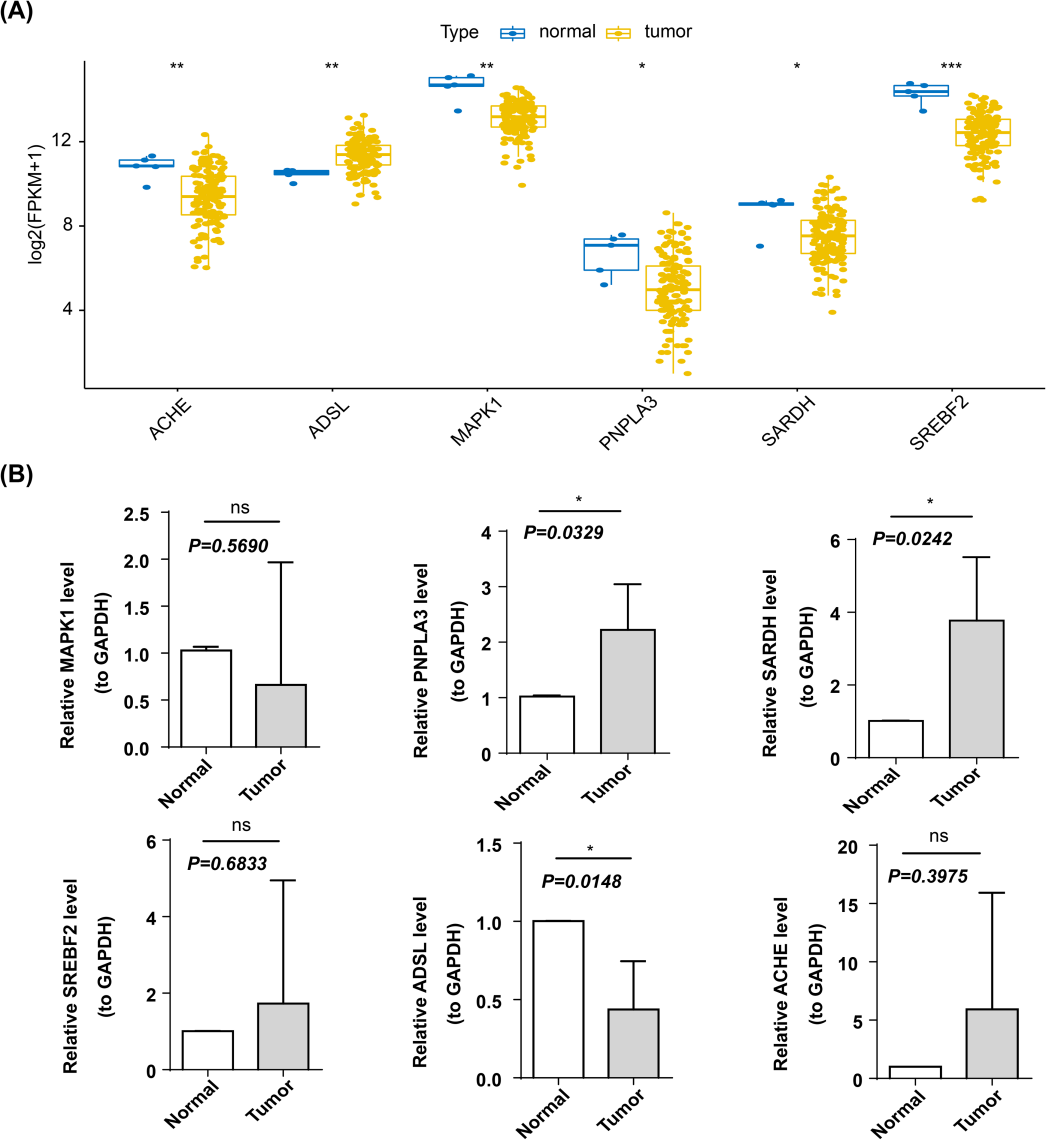
Expression validation of biomarkers. *p < 0.05; **p < 0.01; ***p < 0.001, ns, not significant **(A)** Box line plots of prognostic genes among different groups. Horizontal coordinates represent different prognostic genes, vertical coordinates represent gene expression, and blue dots represent each individual in normal samples and yellow dots represent each individual in cancer samples. **(B)** Expression of prognostic genes in control and tumor samples.

In the TCGA-GBM dataset, the expression levels of six biomarkers were significantly different between GBM and normal groups, and the biomarkers except ADSL were lower in the GBM group (**Figure 10A**). The expression of biomarkers was further confirmed using qRT-PCR. There were no significant differences in the expression levels of the three genes—MAPK1, SREBF2, and ACHE—between the control and tumor groups. In contrast, the expression levels of PNPLA3 and SARDH were significantly elevated in the tumor group compared to the control group, while ADSL expression was notably reduced in tumor tissues (**Figure 10B**).

## 4 Discussion

GBM is a highly aggressive brain tumor characterized by a high recurrence rate and poor prognosis. Despite appropriate treatment, patients experience low five-year survival rates and diminished quality of life (20, 21). As research on GBM deepens, more and more researchers are beginning to pay attention to the role of metabolic reprogramming in GBM (22, 23). It has been shown that dysregulation of propionate metabolism leads to the accumulation of MMA in cells and tumors, which can increase the invasiveness of cancers (10). Malignant tumors frequently exhibit abnormal metabolic activity; however, similar disease phenotypes can arise from different molecular causes. Therefore, categorizing patients based on their molecular profiles could aid in developing more precise treatment prediction models (11, 24). Considering the scarcity of research on whether propionate metabolism disorder affects the progression of GBM, we were inspired by the above study to explore whether such disorder could impact the occurrence and development of GBM. Therefore, we attempted to identify biomarkers associated with propionate metabolism and construct risk models using bioinformatics approaches. Our goal is to provide new insights for the survival assessment and targeted therapy of GBM patients.

This study aimed to develop and validate prognostic indicators for GBM by examining genes related to propionate metabolism. We identified 180 DE-PMRGs between the GBM and control groups. Enrichment analysis revealed that 180 DE-PMRGs were associated with various pathways, including the insulin signaling pathway and the AMPK signaling pathway. Numerous studies indicate that the insulin signaling pathway plays a crucial role in the onset and progression of GBM (25, 26). Insulin and insulin analogues bind to insulin receptor, insulin-like growth factor receptor and hybrid receptors, activating the mitogen-activated protein kinase signaling pathway (MAPK signaling pathway), phosphatidylinositol 3 kinase signaling pathway (PI3K) and other possible signaling pathways in cells, promote cell mitosis, proliferation and anti-apoptosis, and increased risk of tumor formation and metastasis (27, 28). Through further research into the regulatory network of AMP-activated protein kinase (AMPK), it has been found that AMPK may exert dual functions of promoting and inhibiting tumor development through different signaling pathways in different types of tumor cells and specific development stages (29–32). Metformin, a classic anti-diabetic drug and an AMPK activator, has also attracted the attention from the cancer community (33, 34). Based on the enrichment analysis findings, we hypothesize that DE-PMRGs may influence the onset and progression of GBM by modulating or interacting with the insulin and AMPK signaling pathways. These findings also provide further insights into the potential molecular mechanisms of PMRGs in GBM.

In addition, six biomarkers (SARDH, ACHE, ADSL, PNPLA3, MAPK1 and SREBF2) related to propionate metabolism were obtained by further screening. SARDH and ACHE exhibit hazard ratios (HR) greater than 1, while PNPLA3, SREBF2, MAPK1and ADSL consistently display HR values less than 1. In addition, qRT -PCR validation revealed significant differences in the expression of PNPLA3, SARDH, and ADSL in the control and tumour groups. We hypothesize that SARDH was more likely to act as a risk factor in the development of GBM, while ADSL was regarded as protective factors.

The Sarcosine Dehydrogenase (SARDH) gene encodes an enzyme predominantly found in the mitochondrial matrix of the liver. This enzyme plays a crucial role in the metabolism of sarcosine, a derivative of glycine, and is tightly regulated through a functional feedback mechanism (35). A deficiency in sarcosine dehydrogenase results in a condition known as sarcosinemia (36). Recent studies have linked SARDH methylation to tumor growth and invasion. Additionally, overexpression of SARDH in prostate cancer models has been shown to inhibit tumor growth (35, 37). Furthermore, research indicates that SARDH may hinder the onset and progression of colorectal cancer by downregulating specific chemokine genes, namely CXCL1 and CCL20 (38). However, the role of SARDH in GBM has not been clearly reported. The findings of our study suggest that SARDH may potentially serve as a risk factor, and its precise underlying mechanism warrants further investigation.

The nucleotide synthesis pathway can be divided into two types according to the source of substrate: the salvage pathway and the *de novo* pathway. To support unlimited cell proliferation, the metabolic demand for nucleotide biosynthesis in tumor cells increases substantially (39). Adenylosuccinate lyase (ADSL) is an essential enzyme in the *de novo* purine synthesis pathway. The ADSL gene was discovered to have potential role in breast and prostate malignancies early in 1987, and has been found to be up-regulated in a variety of cancer types (40). Recent studies have shown that ADSL promotes the development of prostate cancer by regulating the expression of cell cycle genes (41). In addition, ADSL expression was higher in triple negative breast cancer (TNBC) than in other breast cancer subtypes and normal breast tissues, and ADSL knockout inhibited the proliferation and invasion of TNBC cells both *in vitro* and *in vivo* (42). However, research on the role of ADSL in GBM mechanism is still scarce. Recent research has found that fumaric acid produced by ADSL also plays a role in promoting tumour growth in GBM (43). Through database and *in vitro* validation, we also found that ADSL is differentially expressed in GBM and normal tissues, suggesting that it is a key gene in GBM and that targeting ADSL may affect the development of GBM. However, due to the limited sample size in our experiment, further research needs to be conducted.

In the available studies, patatin-like phospholipase domain-containing protein 3 (PAPLA3) is mainly associated with fat metabolism and has been extensively studied in hepatic diseases such as non-alcoholic fatty liver disease and hepatocellular carcinoma (44–46). The role of PNPLA3 in GBM remains poorly elucidated. In light of our findings, further investigation is warranted to determine whether PNPLA3 functions as a protective factor in GBM.

Cholesterol is a vital component of cell membranes, and sterol regulatory element binding protein 2 (SREBP2) plays a crucial role in maintaining cholesterol balance in the body. SREBP2 is encoded by the SREBF2 gene (47). Abnormal activation of SREBP2 and its downstream target genes can influence the development of various diseases by disrupting cholesterol metabolism. Research indicates that SREBP2 levels are significantly elevated in hepatocellular carcinoma HepG2 cells compared to normal liver cells (LO2) (48). Wen et al. (49) demonstrated that reducing SREBP2 expression modified cellular metabolism in colon cancer, leading to inhibited tumor growth and decreased levels of cancer stem cell-related genes. Additionally, disruptions in cholesterol metabolism can impact the onset and progression of glioblastoma GBM (50). Given its pivotal role in regulating cholesterol metabolism-related genes, SREBF2 is anticipated to be an important target for the treatment of GBM.

Acetylcholinesterase (ACHE) is a key enzyme in biological nerve conduction. At cholinergic synapses, ACHE can degrade acetylcholine, terminate the excitatory effect of neurotransmitters on the postsynaptic membrane, and ensure the normal transmission of nerve signals in the organism. ACHE is involved in cell development and maturation. Moreover, ACHE activity is increased in various primary tumor tissues and in the serum of some cancer patients (51–53). As we shown in the results of the study, ACHE in GBM is more likely to be a risk factor of role. Interestingly, our qRT-PCR results did not support this hypothesis (P>0.05), which we postulated could be attributed to the limited sample size utilized in our study. Consequently, further investigations employing larger sample sizes are warranted.

The mitogen-activated protein kinase 1 (MAPK1, ERK1/2) serves as a dual-functioning kinase and transcription factor in normal physiological conditions, playing crucial roles in proliferation and immunity processes (54). The expression of MAPK1 was significantly upregulated as a tumor promoter in the context of GBM (55). Our study also observed a significant disparity in MAPK1 expression between GBM and normal samples, with MAPK1 exhibiting high levels of expression in CD8 T cells. The serum MAPK1 level can serve as a biomarker in chronic hepatitis B (CHB) to reflect the specific CD8 T cell population (56). The MAPK1 gene has been identified as a crucial regulator of autophagy in the context of acute myocardial infarction, playing a role in modulating CD8 T cells and neutrophils (33). Revisiting the aforementioned studies, we postulate that manipulation of MAPK1 as a means to modulate CD8 T cells could potentially impact the development of GBM.

This study developed a risk model based on the six genes mentioned earlier, revealing a significant difference in survival rates between high-risk and low-risk groups, with the high-risk group showing notably lower survival rates. As a result, an enrichment analysis was performed to explore the biological functions and pathways involved. It was observed that immune response activation and acute inflammatory response were predominantly enriched in terms of biological functions, while cell cycle regulation and ECM-receptor interaction were primarily enriched in pathways. Considering the current state of tumor immune evasion, immunotherapy targeting GBM has been developed to restore anti-tumor immune function within immune cells (57, 58). The research discovered that interactions between tumor cells and ECM receptors promote therapy resistance as well as tumor growth. Targeting these receptors can effectively mitigate radioresistance in GBM (59). The WHO classification of central nervous system tumors has incorporated numerous molecular markers that are closely associated with the diagnosis and prognosis of patients (60, 61). Among these, MGMT. promoter stands out as a significant gene linked to patient prognosis. In this study, we discovered that both the MGMT promoter and risk score can serve as reliable predictors for assessing the prognosis of patients with GBM. This finding provides valuable assistance in diagnosing and prognosticating GBM patients, although further validation through extensive sample verification is still required. Furthermore, a primary limitation of this study is the imbalanced sample size. Future investigations should enhance representativeness by expanding sample sources and incorporating diverse populations. Integrating advanced techniques such as deep learning to uncover hidden associations within the data will offer more robust evidence to advance the understanding of glioblastoma metabolic mechanisms and refine prognostic models.

## 5 Conclusions

This study identified six DE-PMRGs to develop prognostic models that accurately predict outcomes for GBM patients. Furthermore, the risk scores generated from the propionate metabolism-related model correlated with significant biological functions and held clinical importance. This study provides a theoretical basis and reference value for GBM research and treatment in the direction of propionate metabolism. However, this study acknowledges the need for more foundational experiments to support these findings, necessitating additional *in vivo* and *in vitro* research. We will maintain our focus on the six DE-PMRG genes and their ongoing research developments to create personalized treatment strategies for GBM patients.

## Data Availability

Publicly available datasets were analyzed in this study. This data can be found here: TCGA-GBM dataset, Gene Expression Omnibus (GEO); https://www.cancerimagingarchive.net/collection/tcga-gbm/, https://www.ncbi.nlm.nih.gov/geo/.
