# Supplementary figures and images for: Revealing propionate metabolism-related genes in glioblastoma and investigating their underlying mechanisms
Source: Front Oncol. 2025 Apr 17;15:1529369. doi: 10.3389/fonc.2025.1529369 (PMC12043635; doi:10.3389/fonc.2025.1529369)

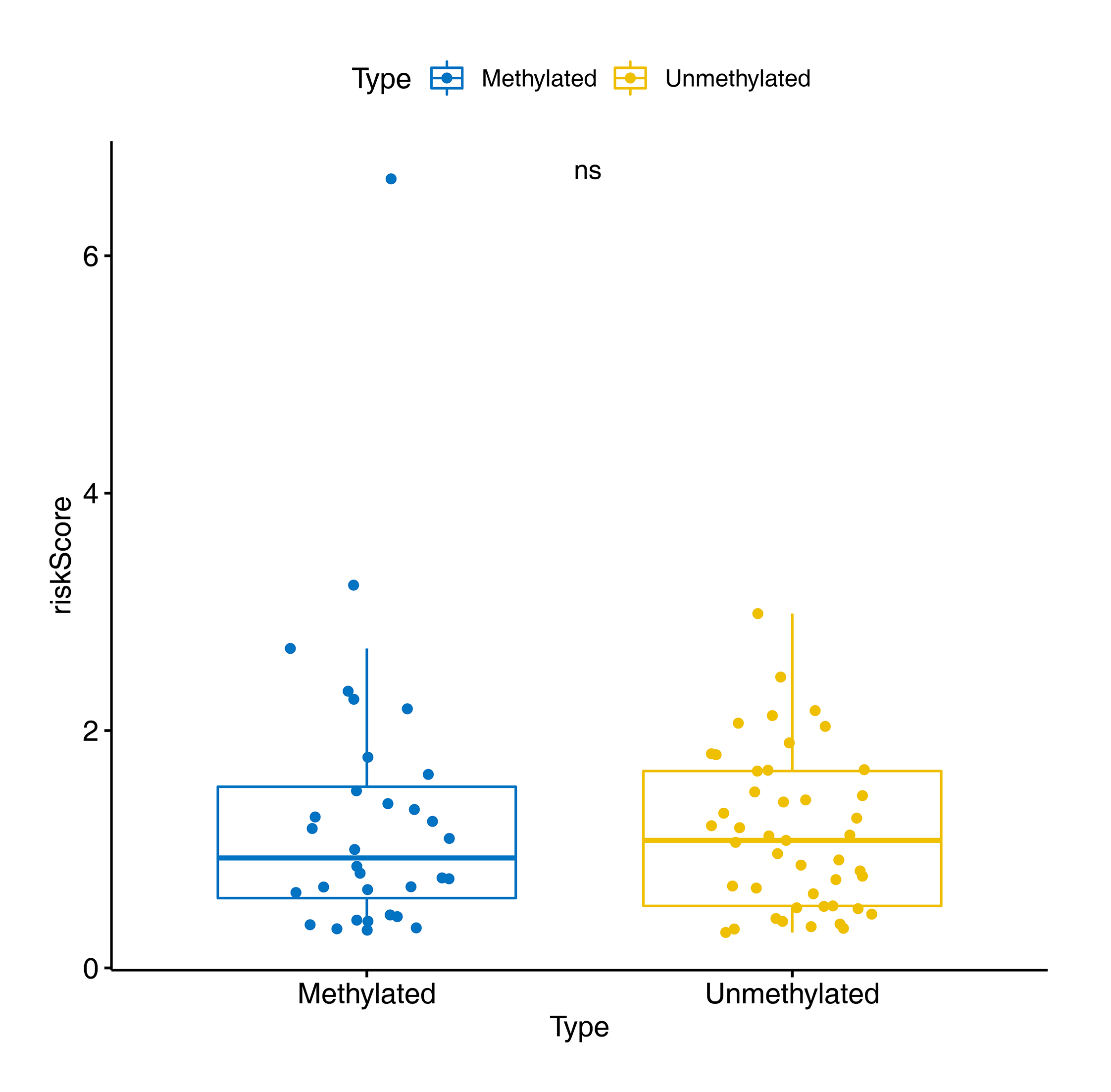

Supplement: Supplementary file 1 [file Image1.tif]

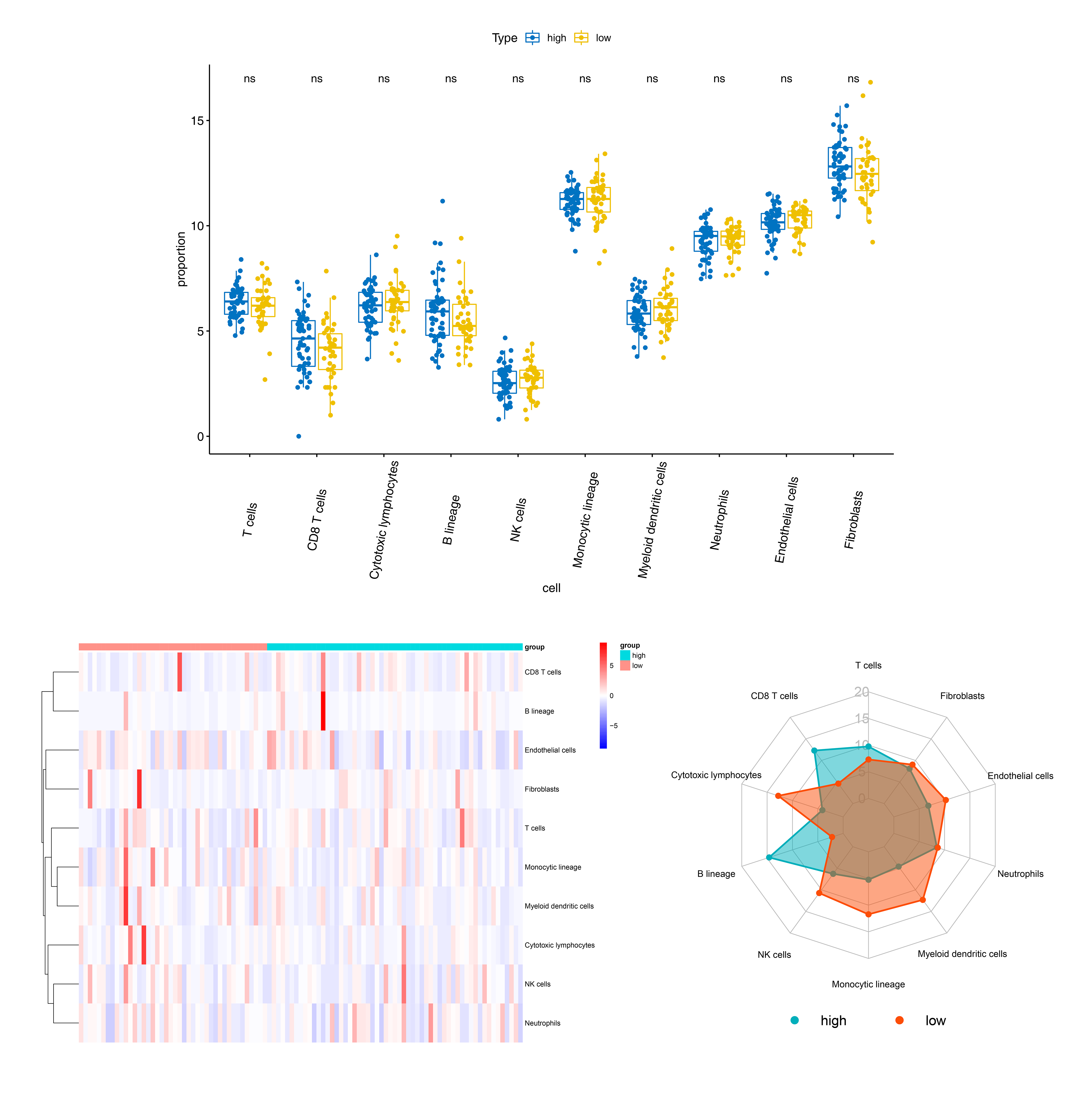

Supplement: Supplementary file 2 [file Image2.tif]

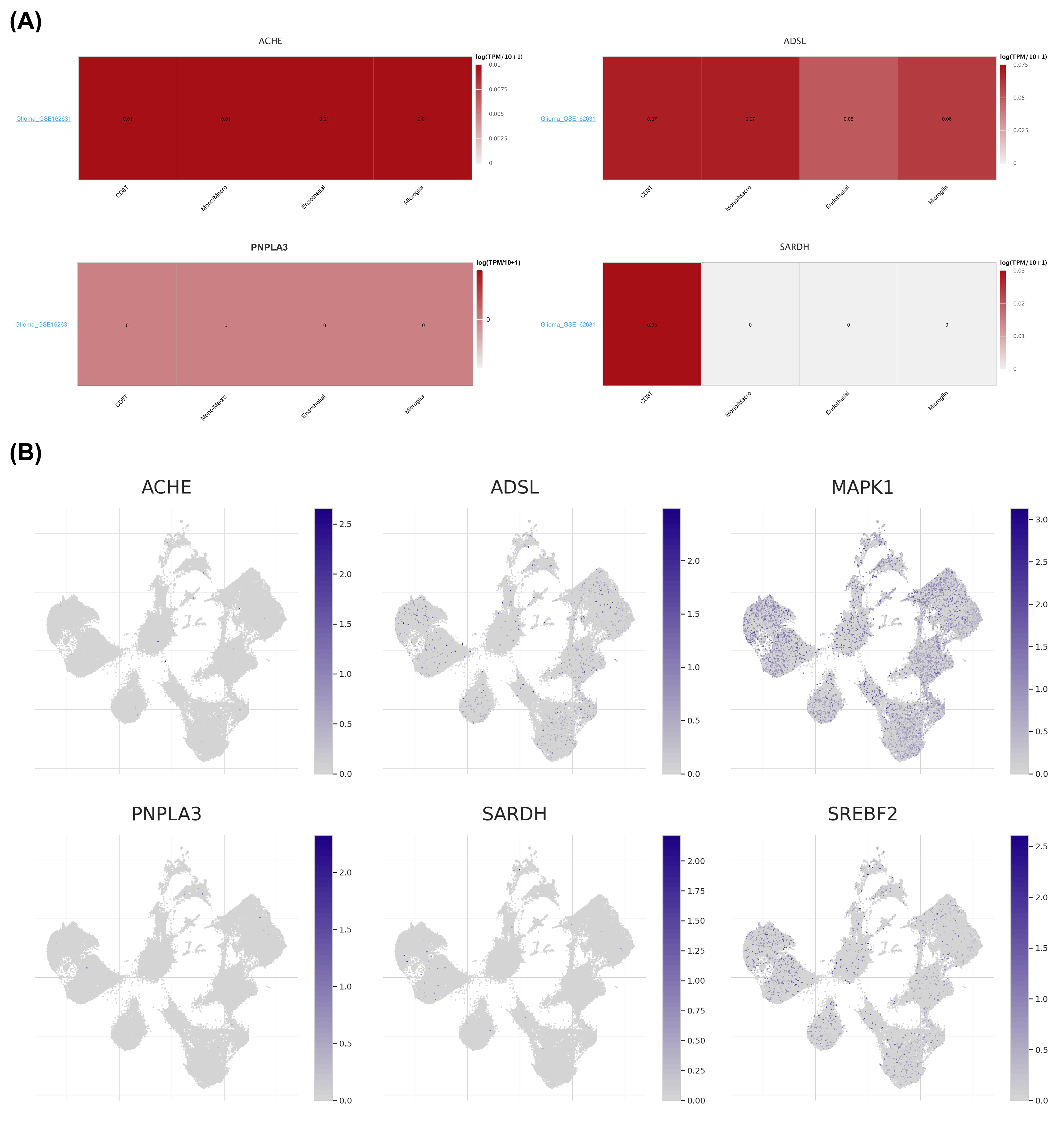

Supplement: Supplementary file 3 [file Image3.tif]
