# Supplementary material for: Revealing propionate metabolism-related genes in glioblastoma and investigating their underlying mechanisms
Source: Front Oncol. 2025 Apr 17;15:1529369. doi: 10.3389/fonc.2025.1529369 (PMC12043635; doi:10.3389/fonc.2025.1529369)
Supplement: Supplementary file 12 [file Table9.docx]

Supplementary Material

## Supplementary Figures


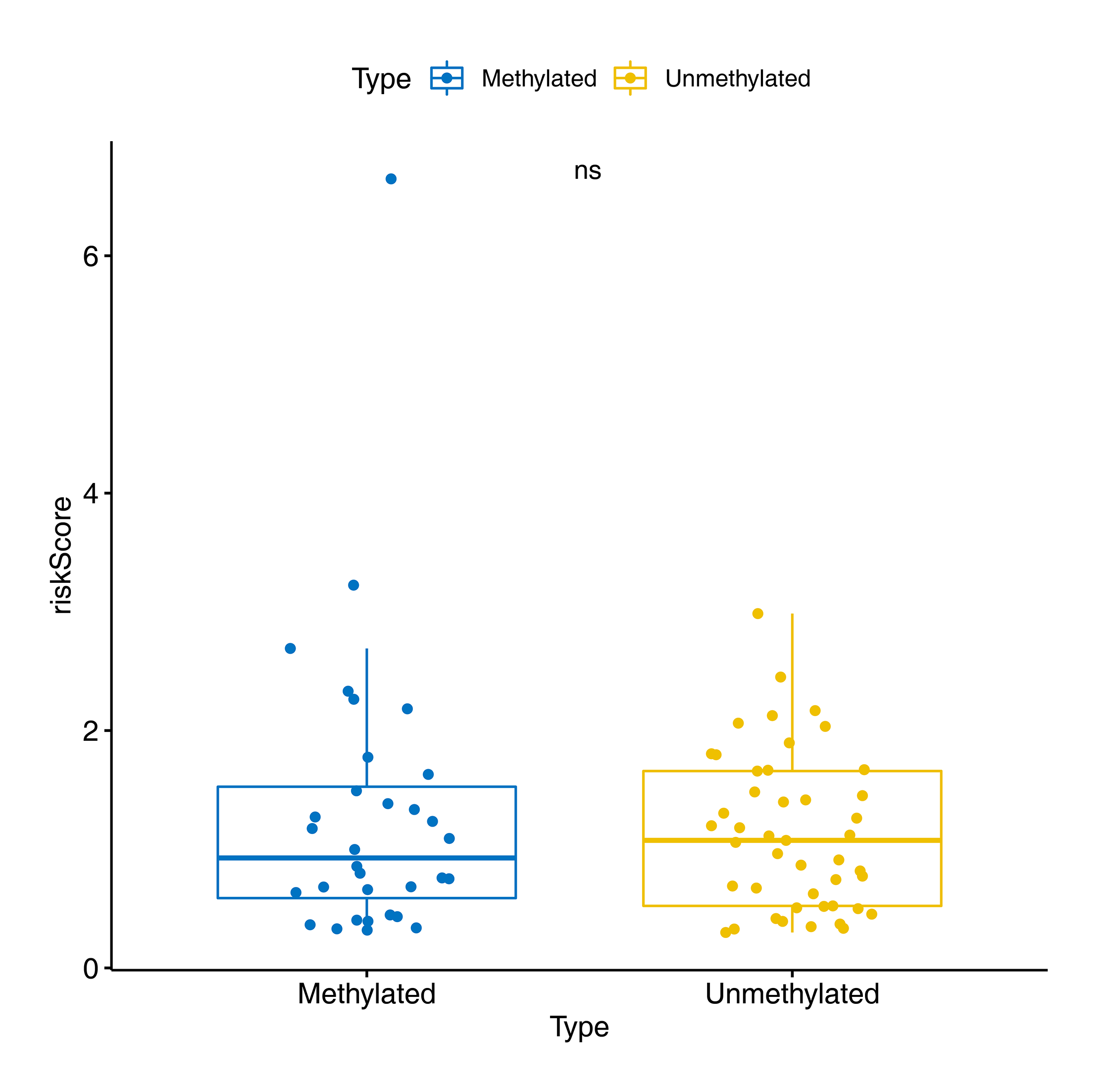


**Supplementary Figure 1.** Correlation of the risk model with MGMT.promoter. where the horizontal coordinate represents the MGMT.promoter grouping and the vertical coordinate represents the value at risk.


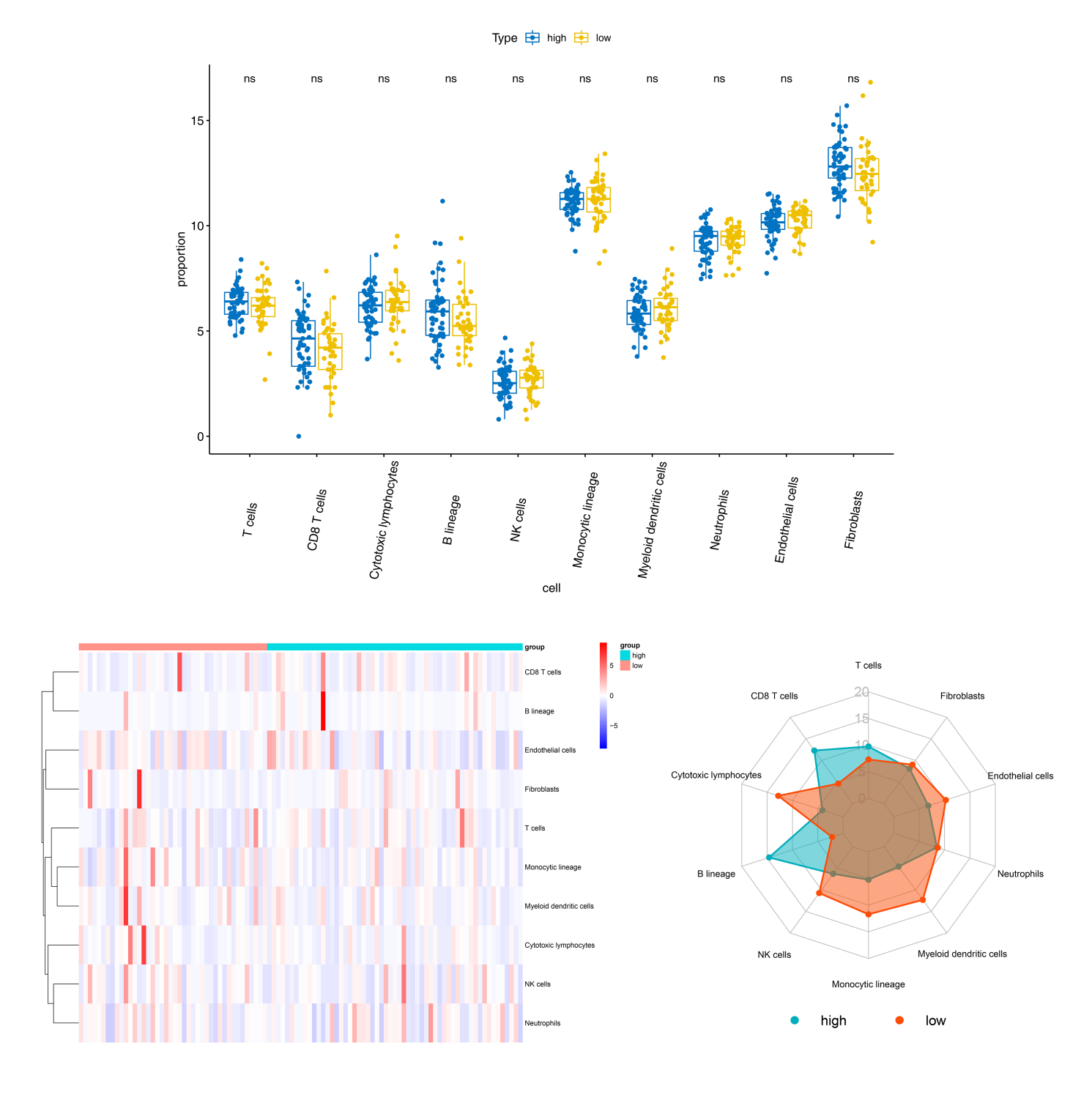


**Supplementary Figure 2.** MCP-counter immune infiltration analysis. (A) Box plots of MCP-counter derived cell content across groups. Yellow color indicates low risk group and blue color indicates high risk group. (B) MCP-counter heatmap for deriving the content of different cells. Each small square represents the content of a different cell in each sample, and its color indicates how much content there is, the more content the redder the color, and the less content the bluer the color. (C) Box plots of MCP-counter derived cell content between groups. Orange color indicates low risk group and blue color indicates high risk group.


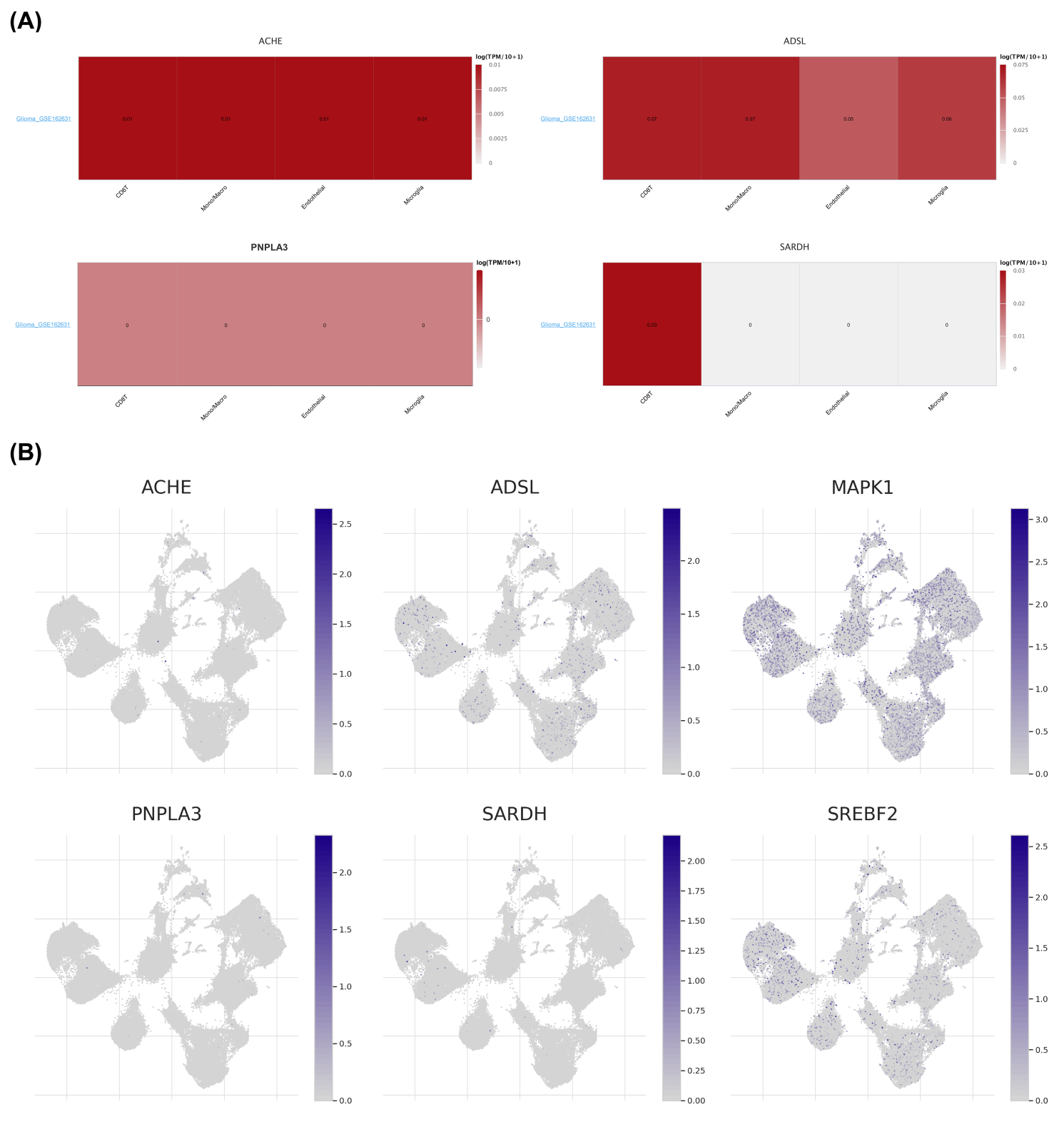


**Supplementary Figure 3.** TISCH single-cell visualization. (A) GSE162631 Heatmap of expression of the single-cell dataset ACHE , ADSL\PNPLA3\SARDH. Redder color indicates higher expression. (B) GSE162631 Single-cell dataset ACHE\ADSL\PNPLA3\SARDH \MAPK1\SREBF2 clustering map. Redder color indicates higher expression.

## Supplementary Tables

## Supplementary Tables 1. Information sheet for 604 PMRGs.

## **Supplementary Tables 2.**The primer information used in Quantitative real time polymerase chain reaction (qRT-PCR).

**Supplementary Tables 3.** Summary of differentially expressed genes (DEGs) between GBM and normal groups.

**Supplementary Tables 4.** Identification of differentially expressed prognostic marker-related genes (DE-PMRGs) from DEGs and PMRGs.

**Supplementary Tables 5.** Gene ontology (GO) molecular function (MF) analysis of DEGs.

**Supplementary Tables 6.** KEGG pathway enrichment analysis results.

**Supplementary Tables 7.** Gene ontology (GO) enrichment analysis of Immune response terms.

**Supplementary Tables 8.** KEGG pathway enrichment analysis results.
